# Evaluating the Reduced Hydrophobic Taste Sensor Response of Dipeptides by Theasinensin A by Using NMR and Quantum Mechanical Analyses

**DOI:** 10.1371/journal.pone.0157315

**Published:** 2016-06-16

**Authors:** Jian Guo, Naoto Hirasaki, Yuji Miyata, Kazunari Tanaka, Takashi Tanaka, Xiao Wu, Yusuke Tahara, Kiyoshi Toko, Toshiro Matsui

**Affiliations:** 1 Department of Bioscience and Biotechnology, Division of Bioresource and Bioenviromental Sciences, Faculty of Agriculture, Graduated School of Kyushu University, 6-10-1 Hakozaki, Fukuoka, 812–8581, Japan; 2 Nagasaki Agriculture and Forestry Technical Development Center, Higashisonogi Tea Research Station, 1414 Nakano-go, Higashisonogi, Nagasaki, 859–3801, Japan; 3 Department of Nutrition, University of Nagasaki, 1-1-1 Manabino, Nagayo, Nagasaki, 851–2195, Japan; 4 Graduate School of Biochemical Sciences, Nagasaki University, 1–14 Bukyo-machi, Nagasaki, 852–8521, Japan; 5 Graduate School of Information Science and Electrical Engineering, Kyushu University, 744 Motooka, Nishi-ku, Fukuoka, 819–0395, Japan; 6 Research and Development Center for Taste and Odor Sensing, Kyushu University, 744 Motooka, Nishi-ku, Fukuoka, 819–0395, Japan; National Research Council of Italy, ITALY

## Abstract

The current study demonstrated that theasinensin A (TSA) had a potential to form the complex with hydrophobic Trp-containing dipeptides, and to reduce their membrane potential by artificial-lipid membrane taste sensor. At a 1:3 molar ratio of the 6 Trp-containing dipeptides together with TSA, we observed a significant chemical shift of the protons of the dipeptides (Δ*δ*) to a high magnetic field, when analyzed using ^1^H-nuclear-magnetic resonance (NMR) spectroscopy. The Δ*δ* values were correlated with the hydrophobicity (log *P*) of the dipeptides and significant correlations were obtained (*P* = 0.022, *R*^2^ = 0.77); e.g., Trp-Leu with the highest log *P* value of 1.623 among the tested dipeptides showed the highest Δ*δ* value of 0.105 ppm for the H7 proton of Trp-Leu, while less chemical shifts were observed in theasinensin B and epigallocatechin-3-*O*-gallate. Diffusion-ordered NMR spectroscopy revealed that the diffusion coefficient of 3 mM of Trp-Leu (7.6 × 10^−11^ m^2^/s) at a pulse field gradient in the range 0.05–0.3 T/m decreased in the presence of 3 mM TSA (6.6 × 10^−11^ m^2^/s), suggesting that Trp-Leu forms a complex with TSA. Quantum mechanical calculations and rotating frame nuclear Overhauser effect-NMR spectroscopy provided configuration information on the geometry of the complex that Trp-Leu formed with TSA (1:1 complex) with a Δ*G* energy of –8.7 kJ/mol. A sensor analysis using artificial-lipid membranes demonstrated that the changes in membrane potential of 1 mM Trp-Leu (21.8 ± 1.3 mV) and Leu-Trp (5.3 ± 0.9 mV) were significantly (*P* < 0.001) reduced by 1 mM TSA (Trp-Leu, 13.1 ± 2.4 mV; Leu-Trp, 3.5 ± 0.5 mV; TSA alone, 0.2 ± 0.01 mV), indicating the effective suppression of hydrophobicity of dipeptides by TSA-formed complex.

## Introduction

Small peptides (di and tripeptides) have long been recognized for their nutritional and functional properties, and are preferable for intestinal absorption rather than amino acids [1,2]. These peptides have antihypertensive [3], vasorelaxant [4], and cholesterol-lowering effects [5]. Irrespective of their health benefits, the poor sensory properties of small peptides owing to their bitterness limits their development for use as advanced functional foods [6]. In order to improve the sensory quality of foods containing small peptides, attempts such as creating physical barriers [7] or masking by flavors [8] and chemicals [9] have been made to reduce the bitterness of amino acids or protein hydrolysates. Taking into consideration that the bitterness of peptides or amino acids is partly associated with their hydrophobicity [10, 11], the bitterness was successfully reduced by using cyclodextrin [12, 13], carrageenan [14], and phospholipids [15]. However, these masking compounds may change the characteristics of the food texture by increasing viscosity.

Theasinensins are oxidative dimers of catechins that possess physiological functions such as anti-hyperglycemic [16, 17] and cholesterol-lowering effects [18]. Besides their physiological function in providing health benefits, we recently reported that theasinensin A (a dimer of (−)-epigallocatechin-3-*O*-gallate (EGCG); abbreviated as TSA) improved the solubility of hydrophobic hesperidin through the formation of a stable complex between TSA and hesperidin in water by hydrophobic interaction [19]. The formation was associated with the symmetric configuration of TSA in a water system by using *in silico* quantum mechanical (QM) analysis [19].

Considering the preference of TSA to form a complex with hydrophobic compounds in water, we investigated the potential of complex formation of TSA with hydrophobic dipeptides in this study. Hitherto, researchers have reviewed the bitterness of peptides by the interaction between molecular property of peptides and G-protein coupled human bitter taste receptors (hTAS2Rs). Maehashi et al. [11] reported that a bitter receptor subtype of hTAS2R1 in cells was stimulated by hydrophobic dipeptides, Gly-Phe and Gly-Leu. Further study by Kohl et al. [20] provided the evidence that the recognitions of hTAS2R subtypes differed by peptide sequence and stereoisomers. Owing to the complex receptor recognition for dipeptides in cell lines, the present study focused on the effect of dipeptide-TSA complex formation on the sensor response in hydrophobic membrane by using a Taste Sensing System that can detect the change in membrane potential by adsorption of hydrophobic targets onto artificial-lipid membranes as a transducer for multichannel taste sensors [21, 22]. In this study, we selected Trp-containing dipeptides because Trp has the highest free energy amongst all amino acids [23], and Trp-containing dipeptides such as Trp-Leu stimulated TAS2Rs in cells, acting as physiologically bitter compounds [20]. In addition, dipeptides containing Trp positioned at the N-terminal were used for this study, as dipeptides containing Trp positioned at the C-terminal showed much lower log *P* values or lower hydrophobicity than that shown by the dipeptides containing Trp at the N-terminal (in Table 1, *e*.*g*., log *P*: Trp-Leu, 1.623; Leu-Trp, 1.019; Trp-Met, 1.000; Met-Trp, 0.380). Investigations on complex formation between dipeptides and TSA were conducted with nuclear magnetic resonance (NMR) spectroscopy and QM calculation.

**Table 1:** Change in chemical shift (Δ*δ*, ppm) of protons of Trp-containing dipeptides in the presence of TSA by ^1^H-NMR analysis at 25°C.

Peptide	Trp-Leu	Leu-Trp	Trp-Met	Trp-Ala	Trp-Tyr	Trp-Asp
**Log *P*** ^a^	**1.623**	**1.019**	**1.004 (0.380)**^b^	**0.25 (-0.353)**^b^	**0.212 (-0.871)**^b^	**-0.321 (-0.532)**^b^
**Δ*δ*, ppm**						
**H7**	**0.105 (TSB: 0.044; EGCG: -0.003)**^c^	**0.097**	**0.114**	**0.087**	**0.099**	**0.085**
**H6**	**0.055 (TSB: 0.027; EGCG: -0.012)**^c^	**0.052**	**0.059**	**0.037**	**0.048**	**0.035**
**H4**	**0.088 (TSB: 0.035; EGCG: -0.004)**^c^	**0.085**	**0.095**	**0.059**	**0.066**	**0.059**

Δ*δ* was the difference in *δ* value of 3 mM dipeptides between in the absence and presence of 9 mM TSA. Target protons were H4, H6, and H7 in indole ring of each dipeptide.

^*a*^ Log *P* value was calculated using a SciFinder Substance Identifier software (https://scifinder.cas.org/scifinder/view/scifinder/scifinderExplore.jsf).

^*b*^ Numbers in parentheses indicate log *P* values of Met-Trp, Ala-Trp, Tyr-Trp, and Asp-Trp with reversed sequence of the corresponding dipeptides.

^*c*^ Numbers in parentheses indicate Δ*δ* values of 3 mM Trp-Leu in the presence or absence of 9 mM TSB or EGCG.

## Materials and Methods

### Materials

Trp-Leu, Leu-Trp, Trp-Met, Trp-Ala, Trp-Tyr, and Trp-Asp were purchased from Bachem AG (Bubendorf, Switzerland). EGCG was purchased from Wako Pure Chemical Industries Ltd. (Osaka, Japan). TSA and theasinensin B (TSB) were prepared as described in our previous report [24]. Briefly, a solution of EGCG incubated with CuCl_2_ and ascorbic acid was applied to a Diaion HP20 column (Mitsubishi Chemical Co., Tokyo, Japan), followed by a stepwise elution with methanol to obtain TSA [24]. TSB was obtained from a fermented green tea product [25]. An internal standard (IS), 3-trimethylsilyl-1-propanesulfonic acid-*d*_*6*_ (DSS-*d*_*6*_, 98.0 atom% D) was obtained from Santa Cruz Biotechnology Inc. (Santa Cruz, TX, USA). Deuterium oxide (D_2_O, 99.8 atom% D) was obtained from Acros Organics (Fair Lawn, NJ, USA). Other reagents were of analytical grade and were used without further purification.

Log *P* value of each dipeptide was obtained by a SciFinder Substance Identifier software (https://scifinder.cas.org/scifinder/view/scifinder/scifinderExplore.jsf).

### ^1^H-NMR measurements

NMR spectra were obtained by an ECS-400 NMR spectrometer (JEOL, Tokyo, Japan) operating at 400 MHz. Samples were dissolved in D_2_O and transferred to a 5 mm NMR tube (Nihonseimitsu Scientific Co., Tokyo, Japan). All spectra were referenced to DSS-*d*_*6*_ at 0 ppm. Auto-shimming for each measurement was performed with a field-gradient shimming at 4 scans and a receiver gain of 20. NMR data acquisition and analysis were performed using Delta software (version 1.1). ^1^H-NMR spectra were acquired by a single pulse sequence under the following conditions: scans, 16; relaxation delay, 15 s; auto-gain and spinning, 12 Hz; operating temperature, 25°C. Change in the chemical shift of protons (Δ*δ*) of 3 mM dipeptides between the absence and presence of 9 mM TSA, was calculated according to the formula: Δ*δ* (ppm) = *δ* (TSA (-))–*δ* (TSA (+)).

The continuous variation method (Job’s plot) [26] was adopted to determine the stoichiometry of the dipeptide-TSA complex formed. According to the Job’s concept [26], the molar fraction (*χ*) of a guest (dipeptide) in a host (TSA)-guest mixture at the maximal point of the plotting parameter (molar concentration of dipeptide × Δ*δ*) against *χ* indicates the stoichiometry of the host-guest complex. Briefly, solutions of dipeptides with different *χ* values of 0.2 to 0.8 against TSA were prepared for the plot, wherease the total molar concentration of the mixture was kept constant at 3 mM. The Δ*δ* value for H4 proton of Trp-Leu as a guest dipeptide in each mixture solution was obtained by ^1^H-NMR measurement.

### DOSY-NMR measurements

Diffusion-ordered (DOSY)-NMR measurements using spin-echo method [27] were performed at 25°C under the following conditions: scan, 128; 90° pulse-width, 9.9 μs; relaxation delay, 20 s; array type, logarithmic; range of pulse field gradient, 0.05–0.3 T/m; point, 8. The diffusion coefficient (*D*, m^2^/s) value was calculated according to previous reports [19, 28] The *D* value of the sample was normalized to that of DSS-*d*_*6*_ (ca. 5 × 10^−10^ m^2^/s) as IS to avoid the change of *D* value by solvent viscosity and temperature [19]. Change in *D* value (Δ*D*) of the dipeptides between the absence and presence of TSA was calculated according to the formula:

$$\Delta D{\;(\text{m}}^{\text{2}}/\text{s})\;=\;D\;(\text{TSA }(-))\;-\;D\;(\text{TSA }(+))\text{.}$$

### ROESY-NMR measurements

Rotating frame nuclear Overhauser effect (ROESY)-NMR spectra were acquired under the following conditions: 90° pulse width, 9.9 μs; mixing time, 0.25 s; X points, 1024; Y points, 512; scans, 24; relaxation delay, 2 s.

### Quantum mechanical (QM) calculations

QM calculations of the dipeptide complex with TSA in water were performed *via* computational simulations. The initial structures of the dipeptide and TSA were constructed by using Chem3D ver.8.0 (Cambridge Science Computing Inc., Cambridge, MA, USA). The atom type and chirality were assigned by Sybyl-X.2.1 (Tripos, Inc., St. Louis, MO, USA). All calculations were performed using Gaussian 09. The structure was optimized using a long-range-corrected density functional, CAM-B3LYP [29], with the cc-pVDZ basis set in the dielectric model of water (polarizable continuum model, PCM) with a dielectric constant of 78.36 [30]. The optimized conformations of the dipeptide and TSA were used to determine the conformation of the complex using Gaussview 5.0. Gibbs free energy (Δ*G*, kJ/mol) of the complex formation was calculated as the intermolecular energy between the dipeptide and TSA.

### Sensor Analysis of dipeptides by a Taste Sensing System

Change in the membrane potential by adsorption of compound onto membrane (CPA, mV) was measured on a commercial taste sensor (Intelligent Sensor Technology Inc., Kanagawa, Japan) consisting of a Taste Sensing System (TS-5000Z) and a sensor electrode for bitter substances (BT0). The BT0 sensor electrode was composed of a lipid/polymer membrane for the sensing part. The output was a change in the membrane potential (vs. Ag/AgCl electrode saturated with 3.3 M KCl). For the sensor experiments, a sample solution was prepared with 30 mM KCl solution containing 0.3 mM tartaric acid. CPA value [31, 32] was used as an index of sensor intensity between dipeptides (or complex with TSA) and lipid/polymer membrane. Measurements were performed five times and the mean of the 4 CPA values, except for the first measurement, was used for obtaining sensory score of the analyte.

### Statistical analysis

Results were expressed as mean ± standard deviation (SD). Statistical difference between two groups was analyzed by Student’s *t*-test, and statistical differences among the groups were analyzed by one-way analysis of variance (ANOVA), followed by *post-hoc* Tukey-Kramer’s *t*-test. A Pearson’s correlation analysis was performed to evaluate the significant correlation of the curve. A *P* value of *<*0.05 was considered statistically significant. All analyses were performed using a Stat View J 5.0 (SAS Institute, Cary, NC, USA).

## Results and Discussion

### ^1^H-NMR analysis of the complex of dipeptides with TSA

An NMR spectroscopy is a conclusive analytical tool for the evaluation of an analyte in its intact form in liquids without any destructive procedures. In addition, it provides useful information on the structure of a complex in host-guest chemistry, e.g., encapsulation of cyclohexylacetic acid with *β*-cyclodextrin [33]. In a previous report [19], an advanced NMR using DOSY technique proved to be an alternative NMR tool for elucidating the complex formation of TSA with hydrophobic hesperidin in a water system by increasing the *D* value in a gradient-pulse field. The favorable complex formation of dimeric EGCG or TSA with hydrophobic compounds [19] led us to investigate whether TSA could act as a suppressor against hydrophobic small peptides, as hydrophobic peptides caused their limited application in the food industry [11, 20].

**Fig 1:**
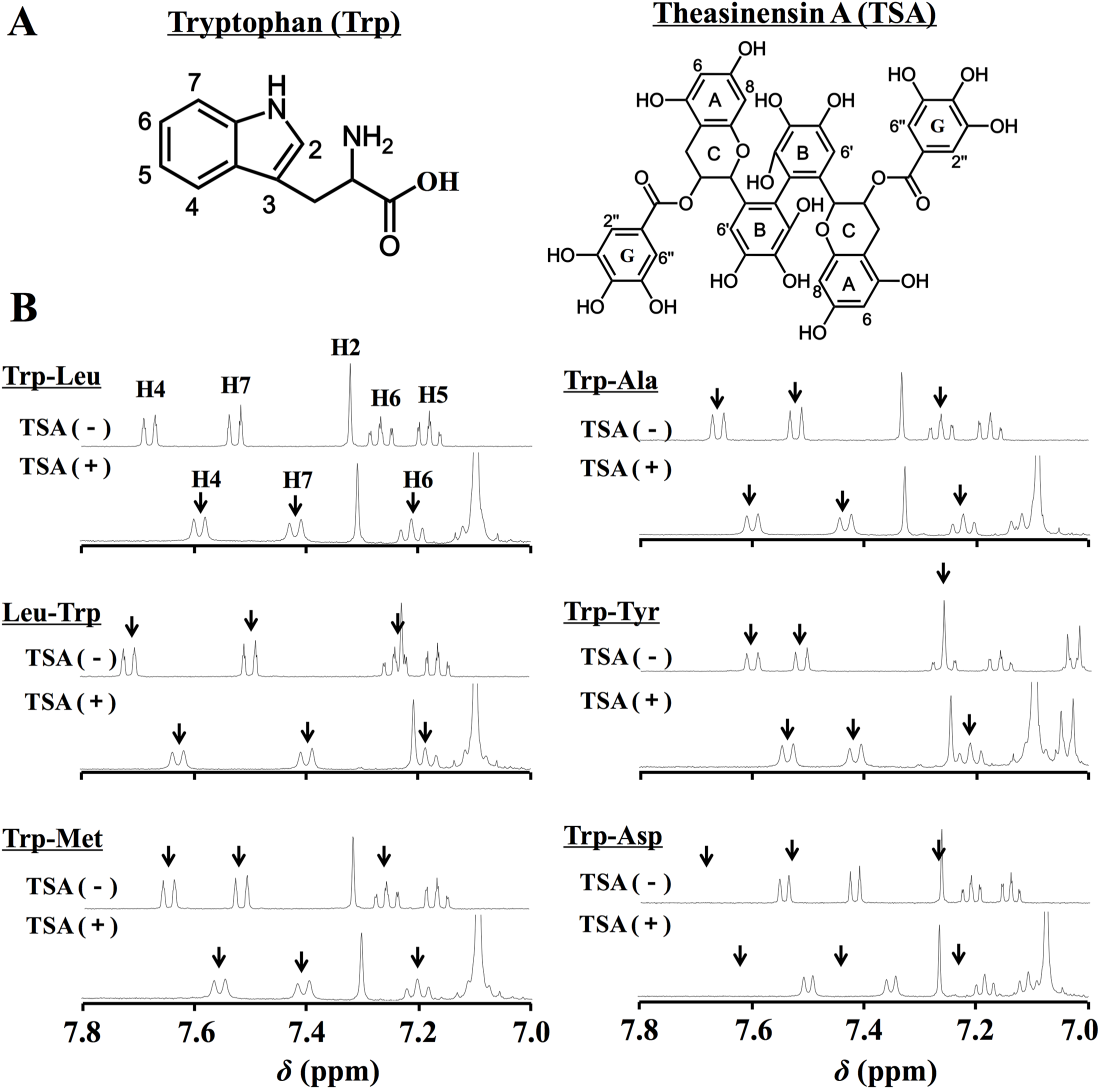
^1^H-NMR spectra of dipeptides in the absence or presence of TSA. (A) Chemical structures of Trp and TSA. (B) ^1^H-NMR spectra (7.0–8.0 ppm) of 3 mM dipeptides in the absence or presence of 9 mM TSA in D_2_O at 25°C. Target protons of each dipeptide with arrow were H7, H6, and H4. The overall views of each spectrum at 0–8 ppm are shown in S1 Fig.

**Fig 2:**
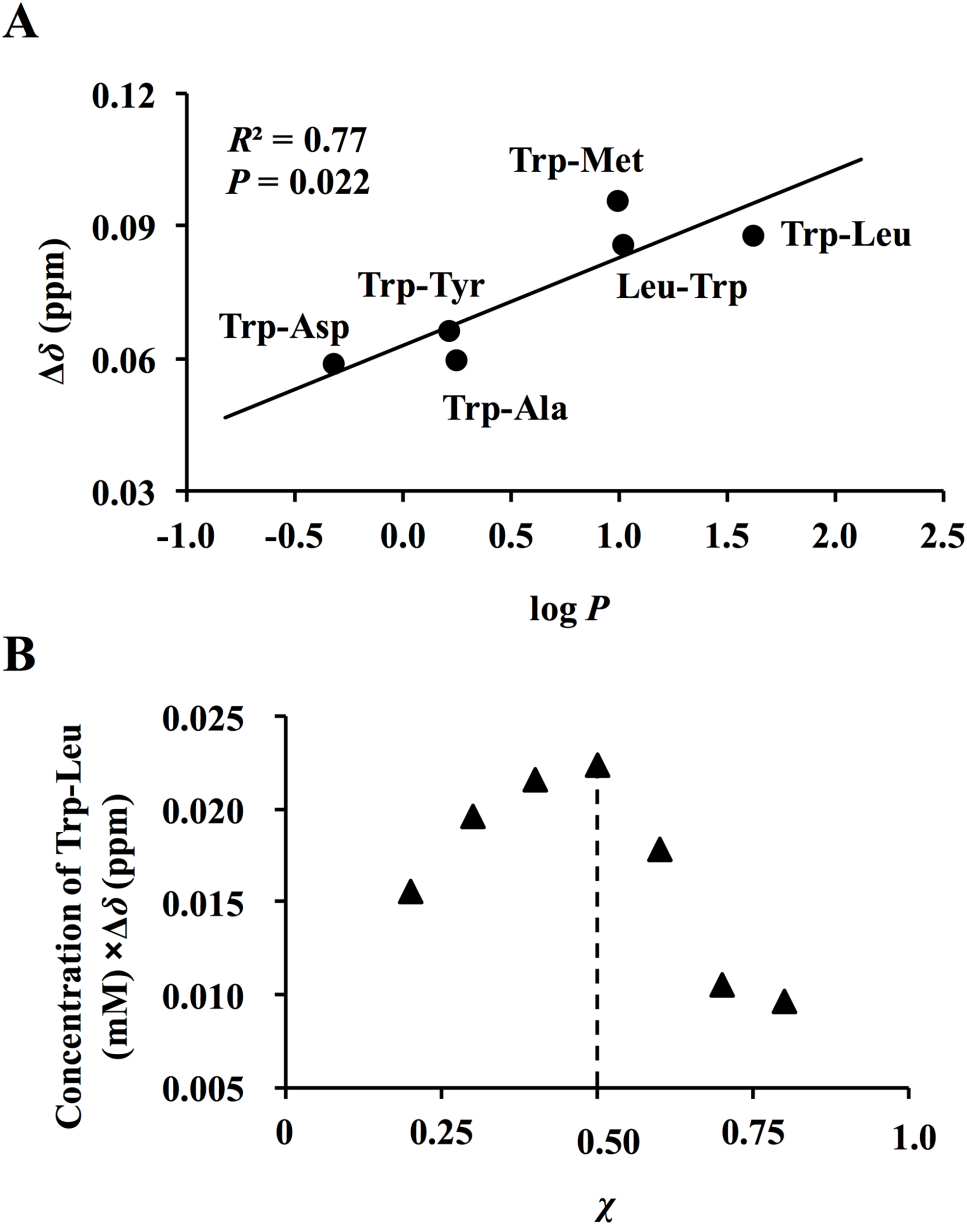
Relationship between log *P* values and the changes in chemical shift of H4 proton (Δ*δ*, ppm) of dipeptides (A), and the continuous variation plot of Trp-Leu with TSA (B). (A) 3 mM dipeptides and 9 mM TSA were used for ^1^H-NMR measurements. The plot was made by the data in Table 1. Target proton for the plot was the H4 in the indole moiety of each Trp-containing dipeptide. (B) Continuous variation plot of Trp-Leu with TSA. Mole fraction (*χ*) of Trp-Leu from 0.2 to 0.8 against TSA was prepared for the plot. Total molar concentration of the mixture of Trp-Leu with TSA was 3 mM. The Δ*δ* value on H4 proton of Trp-Leu in each mixture solution was obtained by ^1^H-NMR measurement in D_2_O at 25°C. Maximal point of the plotting parameter (molar concentration of Trp-Leu × Δ*δ*) against *χ* at 0.5 indicates the stoichiometry of the complex.

Fig 1 shows the ^1^H-NMR spectra of 3 mM Trp-containing dipeptides in the absence or presence of 9 mM TSA at the range of 7.0–7.8 ppm, corresponding to indole protons of Trp (the overall view of each spectrum at 0–8 ppm in S1 Fig). As can be seen in Fig 1B, the 3 protons from the indole ring of Trp in 6 dipeptides (*i*.*e*., H4, H6, and H7 protons; H5 proton was excluded for further experiments due to overlap with a peak from TSA) markedly shifted to a high magnetic field in the presence of TSA, whereas no shifts or less were observed for the other protons, including H2 proton. This clearly indicates that the indole moiety of Trp-containing dipeptides in water was greatly affected or shielded by the TSA molecule. As summarized in Table 1, TSB as well as EGCG (a monomeric catechin of TSA) had no power to induce the proton shifts of dipeptides. The Δ*δ* values of H4, H6, and H7 protons in the indole ring by TSA seemed to be associated with log *P* values of the 6 dipeptides (Table 1). As a function of Δ*δ* for the H4 proton, the relationship between the proton shift and the hydrophobicity of Trp-containing dipeptides was investigated. As depicted in Fig 2A, a significant (*P* = 0.022) relationship between Δ*δ* of the H4 proton and log *P* was obtained with a correlation coefficient (*R*^2^) of 0.77, suggesting that the higher magnetic field shifts of the Trp-containing dipeptides by TSA (Fig 1) were caused by hydrophobic interactions between the dipeptide and TSA in water. Though data are not shown, Δ*δ* values of the H6 and H7 protons also correlated with log *P* (H6: *R*^2^ = 0.71, *P* = 0.0341; H7: *R*^2^ = 0.56, *P* = 0.085). Trp-Leu was used for further consideration on the mechanism of complex formation, because Trp-Leu had the highest log *P* value of 1.623 among the 6 dipeptides and 5 dipeptides with their reversed sequences, including Leu-Trp (log *P*: 1.019), which has been reported as a bitter dipeptide [9].

A prediction of stoichiometry of Trp-Leu-TSA complex was performed by Job’s plot [26]. As shown in Fig 2B, the plot of the molar concentration of Trp-Leu × Δ*δ* for H4 proton revealed a maximal at *χ* of 0.5, indicating that Trp-Leu may form a 1:1 complex with TSA. Therefore, further experiments of dipeptides combined with TSA were performed at a molar ratio of 1:1.

### DOSY-NMR analysis of the complex of dipeptides with TSA

**Fig 3:**
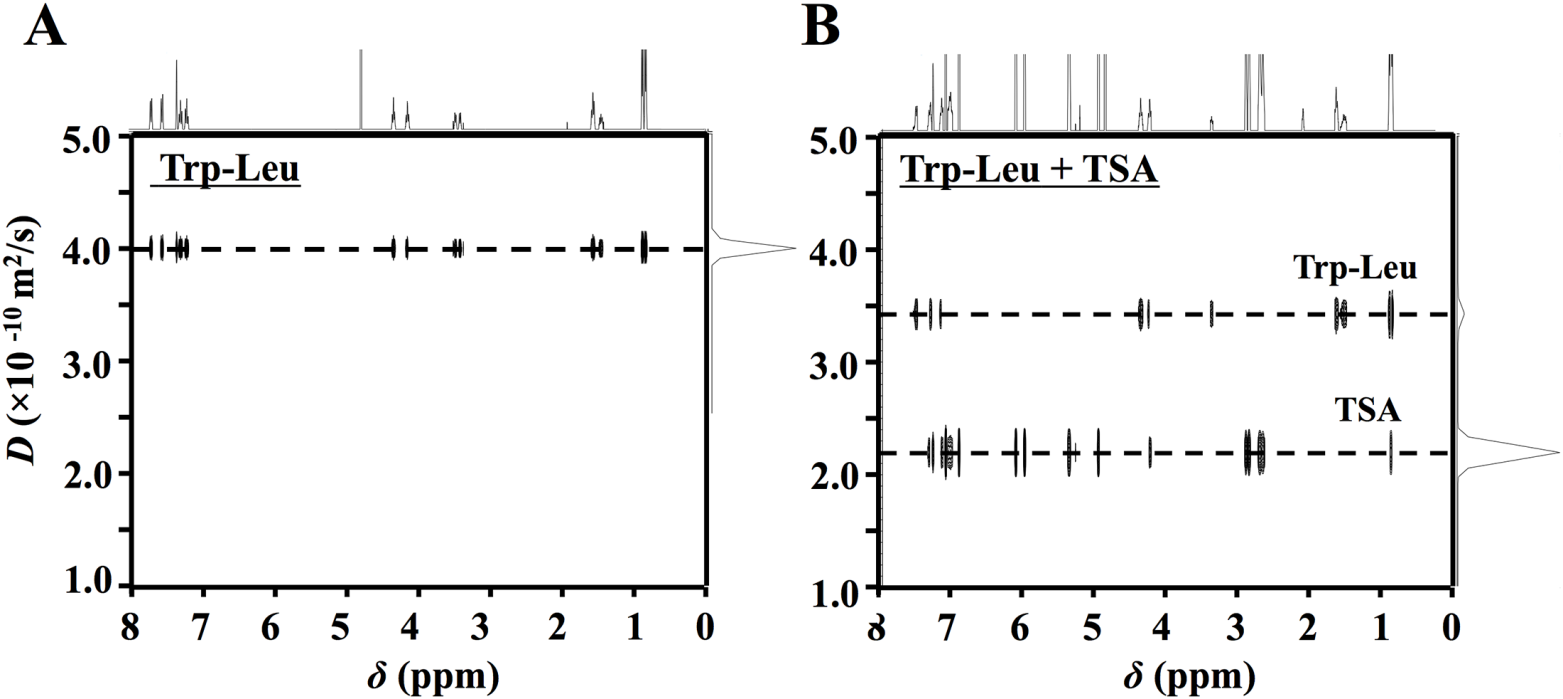
DOSY-NMR spectra of Trp-Leu alone (A) and complex with TSA (B). The spectrum (B) shows a mixture of 3 mM Trp-Leu and 3 mM TSA. DOSY-NMR measurements were performed using the spin-echo method at a field gradient pulse between 0.05 and 0.3 T/m at 25°C.

DOSY-NMR measurements of 3 mM dipeptides in the presence and absence of 3 mM TSA were performed at 25°C. As shown in Fig 3, the 2D-DOSY-NMR spectra of Trp-Leu clearly revealed that the *D* value of Trp-Leu alone (Fig 3A) was reduced in the presence of TSA (Fig 3B); the *D* value of Trp-Leu in the presence of TSA (6.6 × 10−^11^ m^2^/s), which was normalized to that of DSS-*d*_*6*_ as IS, was lower that that of Trp-Leu alone (7.6 × 10−^11^ m^2^/s) (Table 2). Other dipeptides also had a similar tendency; their *D* values, which correspond to molecular size or diffusivity of analyte in a solvent at a field gradient [34], were reduced by TSA (Table 2). These results strongly indicated that an apparent diffusivity of dipeptides in D_2_O was suppressed by any interaction or complex with TSA molecule, similar to a previous report that the *D* value of hesperidin was reduced by TSA with their complex formation in water [19].

**Table 2:** Diffusion coefficients (*D*) of dipeptides in the presence or absence of TSA by DOSY-NMR.

Dipeptide	Trp-Leu	Leu-Trp	Trp-Met	Trp-Ala	Trp-Tyr	Trp-Asp
***D* (TSA (-), × 10** ^**−11**^ **m**^**2**^**/s)**	**7.6**	**7.7**	**8.0**	**7.6**	**7.2**	**8.8**
***D* (TSA (+), × 10** ^**−11**^**m**^**2**^**/s)**	**6.6**	**6.2**	**7.3**	**7.0**	**6.0**	**7.0**
**Δ*D* (× 10** ^**−11**^ **m**^**2**^**/s)**^a^	**1.0**	**1.5**	**0.7**	**0.6**	**1.2**	**1.8**

*D* value was normalized to that of DSS-*d*_*6*_ measured by DOSY-NMR at 25°C.

^a^ Δ*D* was the difference between *D* value of 3 mM dipeptide alone and that in the presence of 3 mM TSA.

### ROESY-NMR analysis of the complex of Trp-Leu with TSA

**Fig 4:**
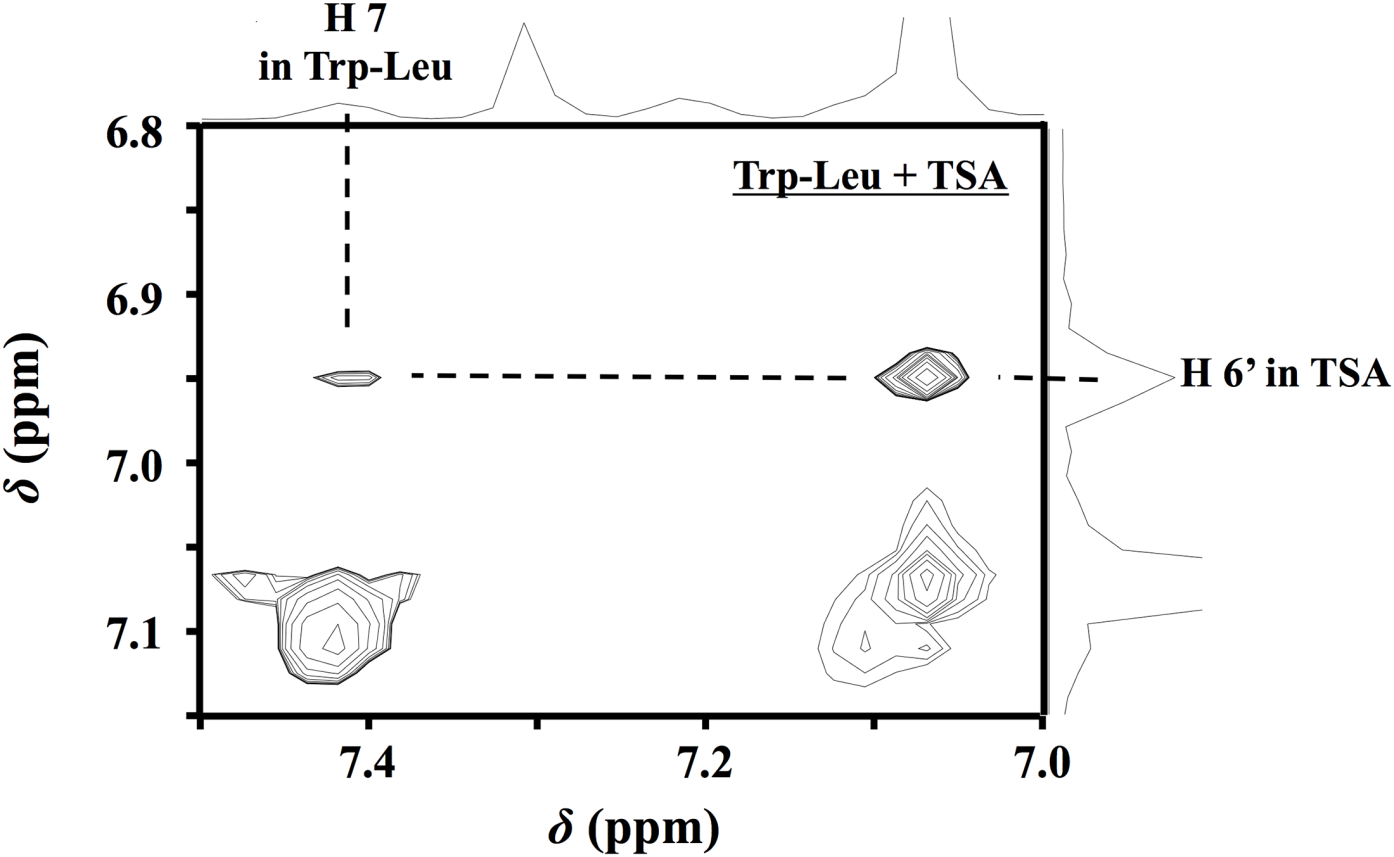
Partial contour plot of the ROESY spectrum of 3 mM Trp-Leu complexed with 3 mM TSA at 25°C.

Configuration analysis of the interaction of Trp-Leu with TSA by ROESY-NMR spectroscopy was performed to get an insight of their stable complex formation in water. As shown in Fig 4, the ROESY spectrum of 3 mM Trp-Leu in the presence of 3 mM TSA gave a correlation cross peak between H7 proton of Trp-Leu and H6’ proton of TSA. This suggested that the hydrophobic indole moiety of Trp-Leu was positioned near the B ring moiety of TSA.

### QM calculation of the complex of Trp-Leu with TSA

**Fig 5:**
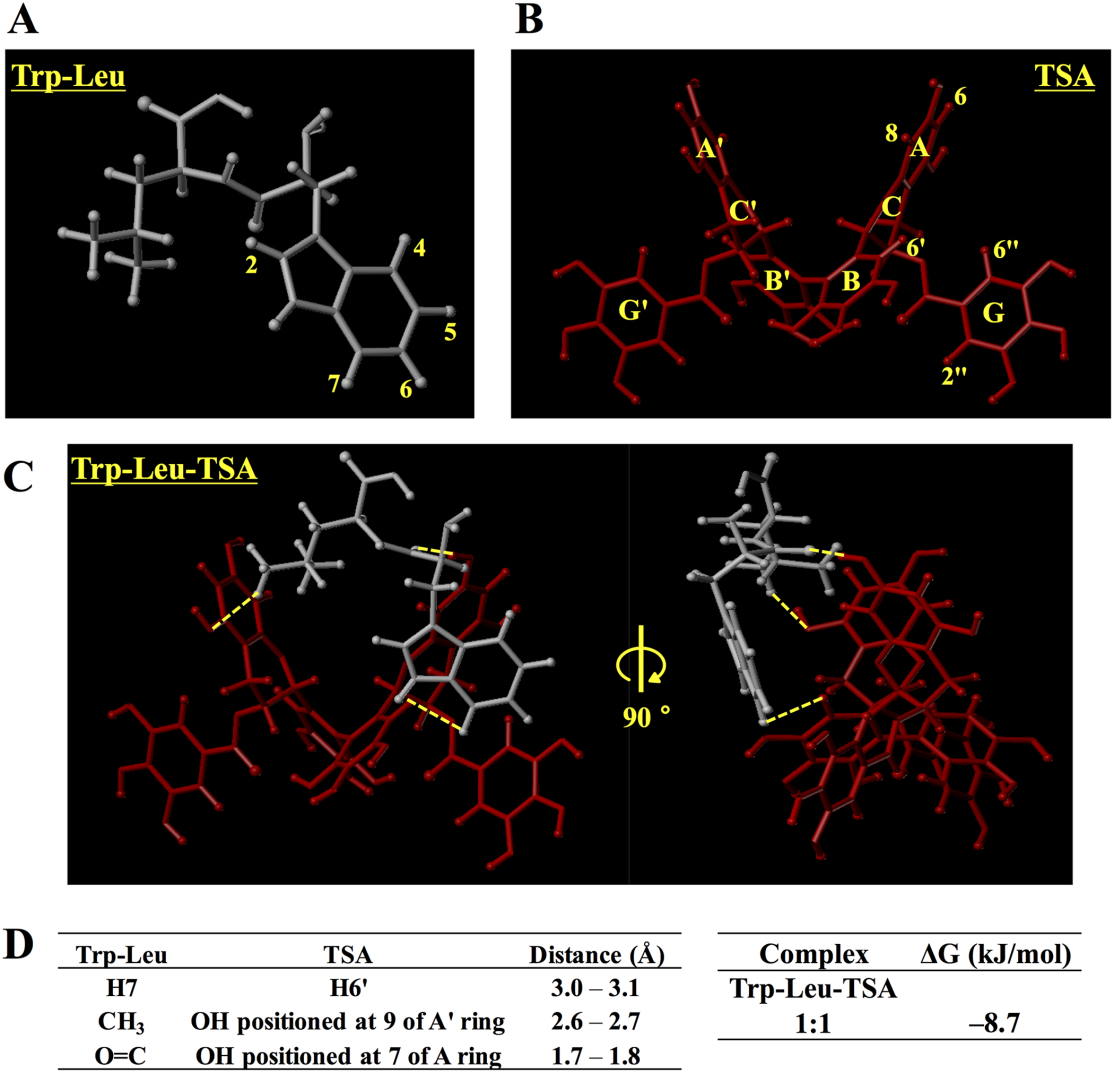
Conformations of Trp-Leu alone (A), TSA alone (B), and the 1:1 complex of Trp-Leu with TSA (C). The interactions between both molecules are indicated by the yellow dotted line. Distance (Å) between Trp-Leu and TSA, and Δ*G* (kJ/mol) of the complex are summarized in (D).

In order to elucidate the steric configuration between Trp-Leu and TSA in the complex, QM calculation for both compounds in virtual water system was performed by computational simulation [19]. As shown in Fig 5A and 5B, Trp-Leu and TSA molecules were successfully optimized in the virtual water system. By fitting each optimized molecule using a GaussView, we got a stable virtual location for both compounds in water with the minimal energy of the formed complex (Δ*G* = − 8.7 kJ/mol) (Fig 5C and 5D). According to the QM analysis of the complex, the preferred location of the indole moiety of Trp-Leu was near the B ring of the symmetric TSA molecule; this was confirmed with the results of ROESY-NMR (Fig 4). In addition, Trp-Leu formed a 1:1 complex with TSA, as predicted from the result in Fig 2B, in which the alkyl side chain of Leu was positioned near the hydrophobic A’ ring of TSA. Regarding the complex formation of polyphenols, it has been reported that gallotannins could interact with the aliphatic side chains of proteins through hydrophobic interaction [35]. In addition, it was reported that EGCG formed a complex with a salivary protein, histatins in a hydrophobic interaction with the basic and aromatic residues of the protein [36]. In addition to the potential of polyphenols on complex formation with proteins, the present study provides further information on polyphenol-aided complex formation by showing that small peptides (in this study, hydrophobic dipeptides) can be stabilized by a symmetric polyphenol, TSA.

### Sensor analysis of dipeptides in the presence of TSA

**Fig 6:**
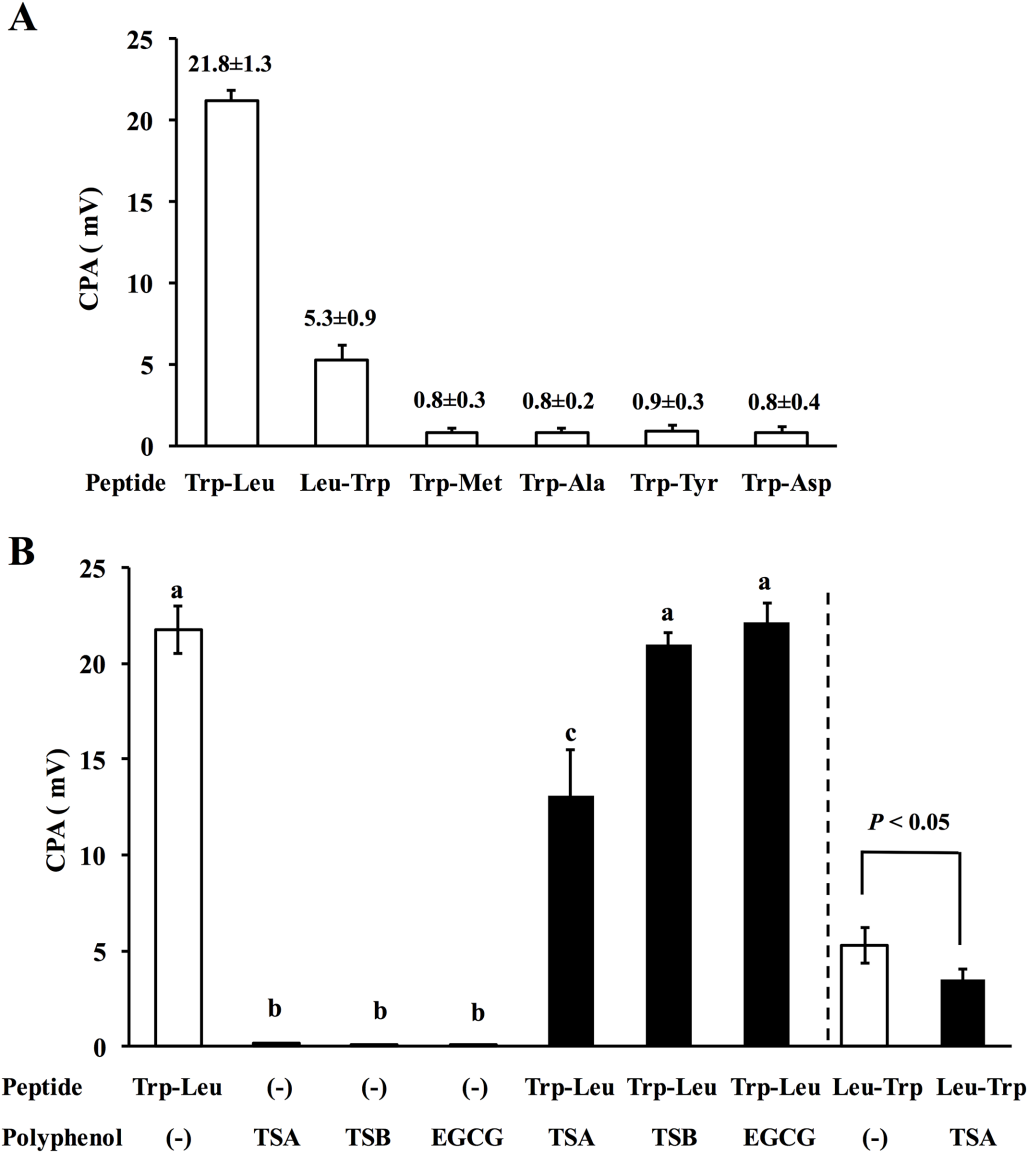
Changes in CPA response of dipeptides in the absence (A) and presence of TSA, TSB, or EGCG (B) by a Taste Sensing System using artificial-lipid membranes. Dipeptides (Trp-Leu, Leu-Trp, Trp-Met, Trp-Ala, Trp-Tyr, and Trp-Asp) and TSA, TSB, or EGCG (each concentration, 1 mM) were used for CPA measurements. CPA (mV), i.e., a change in the membrane potential by adsorption. Results are expressed as the mean ± SD (n = 3). Means without a common letter in all groups differed at *P* < 0.05 by Turkey-Kramer’s *t*-test. Significant difference between two Leu-Trp groups in the absence and presence of TSA was performed by Student’s *t*-test.

By the aid of our developed Taste Sensing System using artificial-lipid membranes [21], a sensory score of 6 dipeptides in the presence or absence of TSA was evaluated by CPA value (mV). Fig 6A shows the CPA values of 6 dipeptides at a concentration of 1 mM with a BT0 lipid/polymer membrane sensor electrode. As a result, the sensor electrode of the Sensor System detected a novel response for Trp-Leu and Leu-Trp with measured CPA values of 21.8 ± 1.3 mV and 5.3 ± 0.9 mV, respectively, while other dipeptides of Trp-Met, Trp-Ala, Trp-Tyr, and Trp-Asp were not responded by the Sensor with less than 1 mV. Considering the significant response by the sensor electrode, further CPA experiments were performed for Trp-Leu and Leu-Trp, showing potent CPA response of >1 mV. TSB (EGCG-epigallocatechin dimer) and EGCG (each 1 mM) were also used for this study. As shown in Fig 6B, 1 mM TSA (EGCG dimer) alone as well as TSB and EGCG alone did not show any CPA response in the System (TSA, 0.22 ± 0.01 mV; TSB and EGCG, <0.1 mV), indicating less hydrophobic interaction of the three catechins alone with the Taste Sensing System. Fig 6B also provided the first finding that TSA can reduce the sensor response of Trp-Leu, in which a significant (*P* < 0.001) CPA reduction of Trp-Leu in the presence of TSA (CPA: 13.1 ± 2.4 mV) was obtained compared to that of Trp-Leu alone (CPA: 21.8 ± 1.3 mV). A significant (*P* < 0.05) CPA reduction by TSA was also obtained for Leu-Trp (CPA: Leu-Trp, 5.3 ± 0.9 mV; Leu-Trp+TSA, 3.5 ± 0.5 mV). The finding that the developed artificial-lipid membrane can respond to the magnitude of the hydrophobicity of analyte [37] allowed us a speculation that the reduced response of Trp-Leu or Leu-Trp by TSA may be caused by the decreased hydrophobicity, probably because of the complex formation with TSA (Fig 5). No significant reduction in CPA value of Trp-Leu by TSB and EGCG (Fig 6B) also revealed that the symmetric structure of TSA (Fig 5) may be crucial to reduce the hydrophobicity of dipeptides in water.

Hitherto, little research has been conducted for the reduction of the bitter taste or hydrophobicity of amino acids or peptides. A successful example was the application of cyclodextrin through which the bitter taste of not only amino acids, peptides, and protein hydrolysates [12, 13], but also that of omeprazole [38] and quinine [39], was reduced by forming inclusion complexes. Moreover, it has been reported that polymeric compounds, such carrageenan [10] and poly-gamma-glutamic acid [40], were effective in reducing the bitter taste, although their underlying mechanism remains unascertained. In this study, we demonstrated that a dimeric catechin, TSA, could be a candidate used in the reduction of hydrophobicity of dipeptides by artificial-lipid membrane taste sensor, for the first time. However, further experiments are necessary to elucidate their potential for altering the taste of peptides by polyphenols by human sensory evaluation and/or TAS2R-expressed cell-line experiments.

## Supporting Information

S1 Fig ^1^H NMR spectra (0–8.0 ppm) of 3 mM Trp-containing dipeptides in the absence or presence of 9 mM TSA. Conditions for ^1^H-NMR measurements are described in the text.
(TIFF)

S2 Fig Relationship between CPA value (mV) by a Taste Sensing System and the concentration of quinine. A good linear relationship between CPA value and logarithmic concentration of quinine (0.05 to 0.112 mM, *R*^2^ = 0.99) was obtained. Results are expressed as the mean ± SD (n = 3).
(TIFF)

## Abbreviations

TSA: theasinensin A
TSB: theasinensin B
EGCG: (−)-epigallocatechin-3-*O*-gallate
DOSY: diffusion-ordered spectroscopy
ROESY: rotating frame Overhauser effect spectroscopy
DSS-*d*_*6*_: 3-trimethylsilyl-1-propanesulfonic acid-*d*_*6*_
D_2_O: deuterium oxide
*D*: diffusion coefficient
QM: quantum mechanical
