# Supplementary figures and images for: Evaluating the Reduced Hydrophobic Taste Sensor Response of Dipeptides by Theasinensin A by Using NMR and Quantum Mechanical Analyses
Source: PLoS One. 2016 Jun 16;11(6):e0157315. doi: 10.1371/journal.pone.0157315 (PMC4911063; doi:10.1371/journal.pone.0157315)

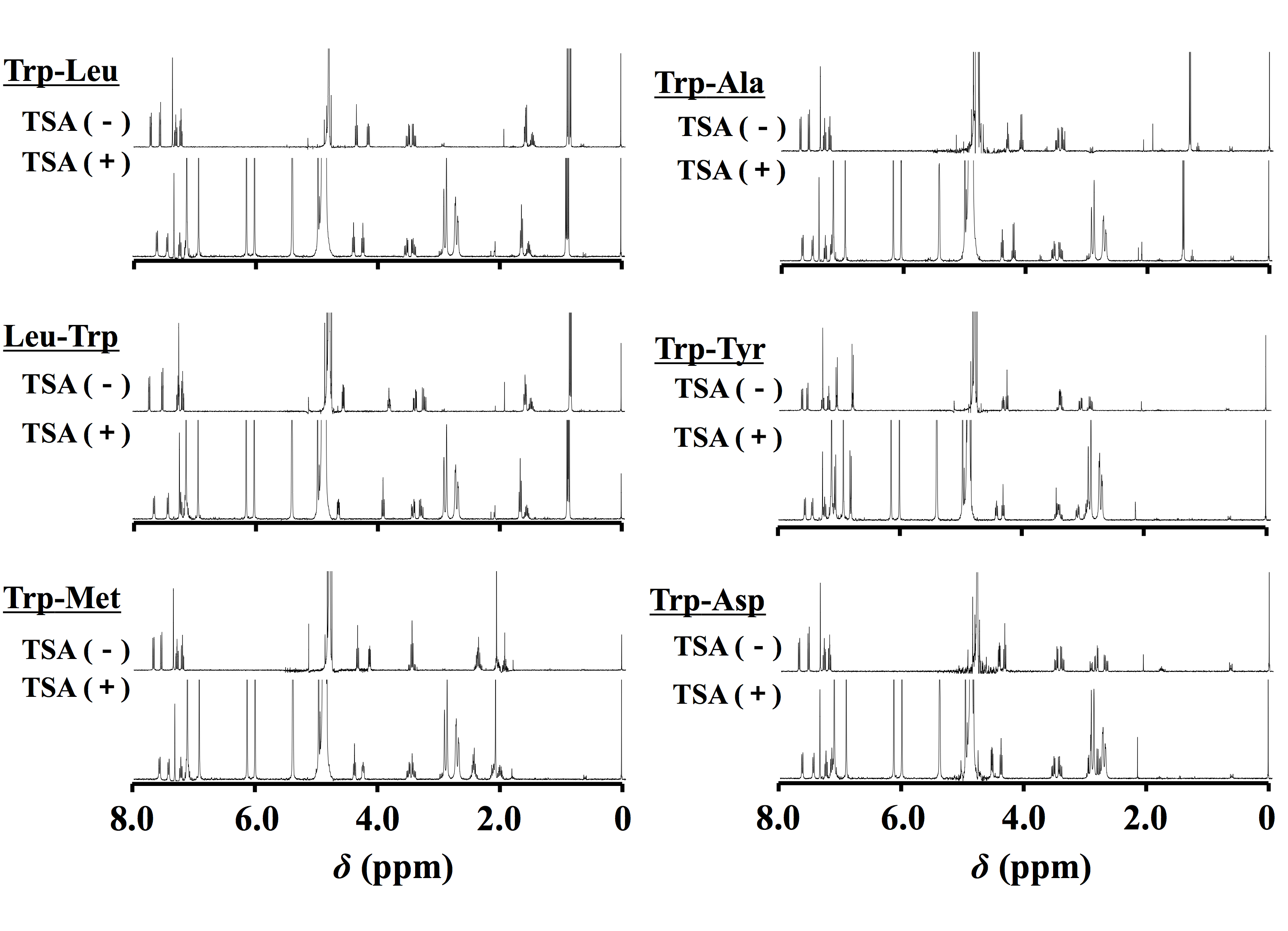

Supplement: S1 Fig — Conditions for 1H-NMR measurements are described in the text. (TIFF) [file pone.0157315.s001.tiff]

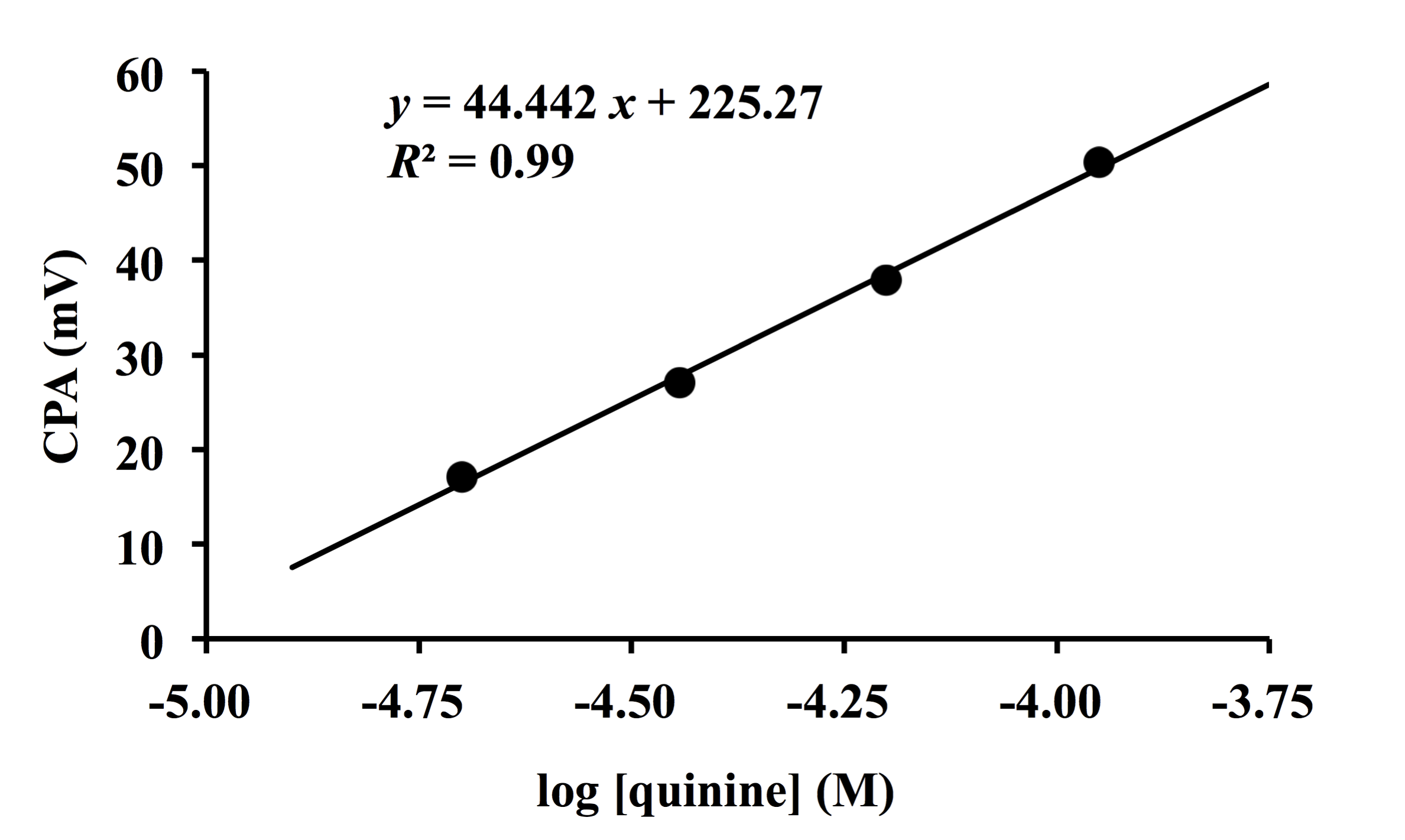

Supplement: S2 Fig — A good linear relationship between CPA value and logarithmic concentration of quinine (0.05 to 0.112 mM, R2 = 0.99) was obtained. Results are expressed as the mean ± SD (n = 3). (TIFF) [file pone.0157315.s002.tiff]
